# microRNA-342-3p targets FOXQ1 to suppress the aggressive phenotype of nasopharyngeal carcinoma cells

**DOI:** 10.1186/s12885-018-5225-5

**Published:** 2019-01-24

**Authors:** Zheqing Cui, Yulin Zhao

**Affiliations:** Department of Rhinology, First Affiliated Hospital of Zhengzhou University, Zhengzhou, China

**Keywords:** Invasion, FOXQ1, Growth, miR-342-3p, Nasopharyngeal carcinoma

## Abstract

**Background:**

microRNA (miR)-342–3p is frequently dysregulated in human cancers. In the present study, we aimed to explore the expression, prognostic significance, and biological relevance of miR-342-3p in nasopharyngeal carcinoma (NPC).

**Methods:**

We examined miR-342-3p expression in 79 paired NPC specimens and corresponding normal tissues and analyzed its prognostic impact on overall survival of NPC patients. Gain- and loss-of-function experiments were conducted to determine the biological roles of miR-342-3p.

**Results:**

Compared with matched normal nasopharyngeal tissues, miR-342-3p was significantly downregulated in NPC (*P* = 0.0038). Low miR-342-3p expression was significantly correlated with reduced overall survival (*P* = 0.0084). Ectopic expression of miR-342-3p significantly suppressed proliferation, colony formation, and invasion of NPC cells. In contrast, depletion of miR-342-3p facilitated NPC cell proliferation and invasion. In vivo xenograft studies confirmed that overexpression of miR-342-3p restrained the growth of NPC xenograft tumors. Mechanistically, FOXQ1 served as a functional target of miR-342-3p. There was a significantly negative correlation between miR-342-3p and FOXQ1 expression (*r* = − 0.487, *P* = 0.004) in NPC specimens. Overexpression of FOXQ1 rescued the inhibitory effects of miR-342-3p on NPC cell growth and invasion.

**Conclusions:**

miR-342-3p downregulation predicts poor prognosis in NPC patients and shows suppressive activity against NPC growth and invasion through repression of FOXQ1. Restoration of miR-342-3p may represent a potential therapeutic strategy for NPC.

## Background

Nasopharyngeal carcinoma (NPC) is a common tumor of the head and neck in Southeast Asia and China [1]. Radiotherapy in combination with chemotherapy is the primary treatment for locally advanced NPC. Due to high recurrence and metastasis rates, the prognosis of advanced NPC is still unsatisfactory, with a 5-year survival rate of approximately 50% in Chinese population [2, 3]. However, the exact mechanism for NPC development and progression has not yet been clarified.

Forkhead box (FOX) proteins are a superfamily of transcription factors that are evolutionarily conserved in eukaryotic organisms [4]. Deregulation of FOX proteins has been linked to tumorigenesis, metastasis, and drug resistance [5]. For example, FOXO1 phosphorylation overcomes chemoresistance in cancer cells by blocking IQGAP1-MAPK interactions [6]. FOXR2 is required for the growth and spread of prostate cancer cells [7]. Targeted reduction of FOXM1 was found to exert suppressive effects on NPC growth [8]. FOXC2 overexpression induces chemoresistance in NPC cells [9]. FOXQ1 acts as an oncogene in NPC [10]. These studies suggest FOX proteins as promising targets for anticancer treatments.

microRNAs (miRs) are a class of short non-coding RNAs that post-transcriptionally regulate gene expression through base pairing to partially complementary sites in the 3′-untranslated region (3′-UTR) of target mRNAs [11]. miRs play a critical role in the growth and progression of cancers [12]. For instance, it was reported that miR-519 inhibits the proliferation of NPC cells by repressing URG4/URGCP [13]. miR-342-3p is frequently dysregulated in human cancers, such as colon cancer [14] and pancreatic cancer [15]. This miR shows growth-suppressive activity in hepatocellular carcinoma [16], lung cancer [17], and gallbladder cancer [18]. miR expression profiling of NPC has revealed the downregulation of miR-342-3p in NPC [19]. However, the biological function of miR-342-3p in NPC is still unknown.

In the present study, we collected 79 paired NPC tissue specimens and adjacent normal tissues and measured the expression of miR-342-3p. The prognostic impact of miR-342-3p was evaluated. To dissect the biological roles of miR-342-3p, gain- and loss-of-function experiments were carried out.

## Methods

### Tissue specimens

We collected 79 paraffin-embedded NPC specimens and their adjacent normal tissues between 2002 and 2008 from the First Affiliated Hospital of Zhengzhou University (Zhengzhou, China). All cases were confirmed by pathological examination. Follow-up data were retrieved from medical records. No patient received anticancer treatment prior to biopsy. There were 51 males and 28 females, with a median age of 56 years (range, 37–85 years). Clinicopathologic characteristics of the patients are shown in Table 1. Written informed consent for research was obtained from each patient. This retrospective study was approved by the Institutional Human Experiment and Ethics Committee of the First Affiliated Hospital of Zhengzhou University.

**Table 1:** Clinicopathological parameters of NPC patients (*n* = 79)

Variable	No. of cases
Age (years)	
> 50	47
≤ 50	32
Gender	
Male	51
Female	28
Clinical stage	
I/II	24
III/IV	55
Lymph node status	
Absent	19
Present	60

### Cell culture

Four human NPC cell lines (C666, SUNE1, HNE1 and CNE2) and NP69 immortalized nasopharyngeal epithelial cells were obtained from the Shanghai Institutes for Biological Sciences, Chinese Academy of Sciences (Shanghai, China). Cells were cultured in RPMI 1640 medium (Thermo Fisher Scientific, Waltham, MA, USA) supplemented with 10% fetal bovine serum (FBS; Sigma-Aldrich, St. Louis, MO, USA). The identity of the cell lines have been validated by short-tandem repeat analyses. They are free of mycoplasma contamination.

### RNA extraction and real-time PCR analysis

Total RNA was extracted from tissues and cells using TRIzol reagent (Invitrogen, Grand Island, NY, USA). Reverse transcription was performed using the TaqMan Reverse Transcription Kit (Applied Biosystems, Carlsbad, CA, USA) and miR-342-3p specific stem-loop primer. Real-time PCR was conducted using the TaqMan MicroRNA Assay Kit (Applied Biosystems) according to the manufacturer’s instructions. U6 was used an endogenous control. The expression of miR-342- 3p was normalized against U6.

### Stable expression of miR-342-3p in NPC cells

To generate miR-342-3p stable clones, C666 and CNE2 cells were transfected with a miR-342-3p-expressing plasmid (Cell Biolabs, San Diego, CA, USA) using Lipofectamine 2000 Reagent (Invitrogen). Control cells were transfected with the pEP-miR Null vector (Cell Biolabs). Forty-eight hours post-transfection, cells were selected in the presence of 2 μg/mL puromycin (Sigma-Aldrich) for 10 days.

### Transient transfection

C666 and CNE2 cells were transfected with anti-miR-342-3p inhibitor or negative control inhibitor (50 nM; Exiqon, Vedbaek, Denmark) using Lipofectamine 2000 Reagent. In some experiments, a FOXQ1-expressing plasmid or empty vector was transfected to C666 cells with stable expression of miR-342-3p. The FOXQ1-expressing plasmid was generated by inserting a full-length open reading frame of *FOXQ1* (lacking the 3′-UTR) into pcDNA3.1(+) expression vector. Twenty-four hours after transfection, cells were tested for gene expression, proliferation, and invasion.

### Cell proliferation and colony formation assays

Cells were seeded onto 96-well plates (4 × 10^3^ cells/well) and cultured for 3 and 5 days. Each well was added with 3-[4,5-dimethyl-2-thiazolyl]-2,5-diphenyl-2H-tetrazolium bromide (MTT) solution (0.5 mg/ml, Sigma-Aldrich) and incubated for 4 h at 37 °C. Dimethyl sulfoxide was used to dissolve the resultant crystals. Absorbance was recorded at 570 nm. For evaluation of colony formation capacity, cells were plated onto 6-well plates (500 cells/well) and cultured for 10 days. After staining with 1% crystal violet (Sigma-Aldrich), the number of colonies was determined using a microscope.

### Transwell invasion assay

We used the methodology previously described by Zhao and colleagues [20]. Cells in serum-free medium containing 10 μg/ml mitomycin C (Sigma-Aldrich) were seeded in the upper chamber of 24-well Transwell plates (2 × 10^4^ cells/well). The inserts (8 μm in pore size) were precoated with Matrigel (BD Biosciences, San Jose, CA, USA). The lower chamber was filled with RPMI 1640 medium containing 10% FBS. The cells were allowed to invade through the Matrigel-coated inserts for 24 h. Afterwards, invaded cells were stained with 1% crystal violet and counted under a microscope.

### Dual-luciferase reporter assay

Luciferase reporter assay was performed as previously described [20]. The 3’-UTR of *FOXQ1* mRNA was inserted into the pMIR-REPORT Luciferase miRNA Expression Reporter Vector (ThermoFisher Scientific, Waltham, MA, USA). A mutant form with disruption of the miR-342-3p binding site was constructed using the QuikChange site-directed mutagenesis kit (Stratagen, Santa Clara, CA, USA). C666 cells were co-transfected with the reporter constructs together with the miR-342-3p-expressing plasmid or empty vector. Luciferase activities were measured using the Dual-Luciferase Reporter Assay System (Promega, Fitchburg, WI, USA) 48 h after transfection. The relative luciferase activity was determined after normalization against *Renilla* luciferase activity.

### Tumorigenic studies in nude mice

For tumorigenic studies, C666 and CNE2 cells with stable overexpression of miR-342-3p or empty vector (2 × 10^6^ cells/mouse) were subcutaneously inoculated into the flanks of male BALB/c nude mice (5 weeks of age; Shanghai Laboratory Animals Center of Chinese Academy of Sciences, Shanghai, China). Tumor growth was monitored for 5 weeks. After the last measurement of tumor volume, animals were are euthanized by carbon dioxide inhalation followed by cervical dislocation. The xenograft tumors were resected and weighed. Afterwards, tumor samples were fixed, embedded in paraffin, and subjected to immunostaining for Ki-67 using an anti-Ki-67 monoclonal antibody (Thermo Fisher Scientific; 1:400 dilution). The experiment protocol was approved by the Animal Care and Use Committee of Zhengzhou University.

### Western blot analysis

Protein samples from NPC tissues and cells were prepared in radioimmunoprecipitation assay buffer (Sigma-Aldrich) supplemented with the Protease and Phosphatase Inhibitor Cocktail (Abcam, Cambridge, UK). Protein samples were separated by SDS-polyacrylamide gel electrophoresis and transferred onto polyvinylidene difluoride membranes. Membranes were probed with rabbit anti-FOXM1 polyclonal antibody (Abcam; 1:500 dilution), anti-FOXQ1 polyclonal antibody (Abcam; 1:500 dilution), or anti-β-actin polyclonal antibody (Thermo Fisher Scientific; 1:5000 dilution), followed by incubation with horseradish peroxidase-conjugated goat anti-rabbit IgG (Sigma-Aldrich). Signals were visualized by enhanced chemiluminescence (Thermo Fisher Scientific). Protein bands were quantified by densitometric analysis using the Quantity One software (Bio-Rad Laboratories, Hercules, CA, USA).

### Statistical analysis

Data are expressed as the mean ± standard deviation (SD). Differences in the means were determined using the Student’s *t* test or one-way analysis of variance (ANOVA) followed by Tukey’s multiple comparison test. The correlation between miR-342-3p and FOXQ1 protein levels in NPC specimens was determined by Pearson’s correlation coefficient analysis. Survival curves were plotted using the Kaplan-Meier method and compared using the log-rank test. *P* < 0.05 was considered statistically significant.

## Results

### miR-342-3p is downregulated in NPC and predicts poor prognosis

**Fig. 1:**
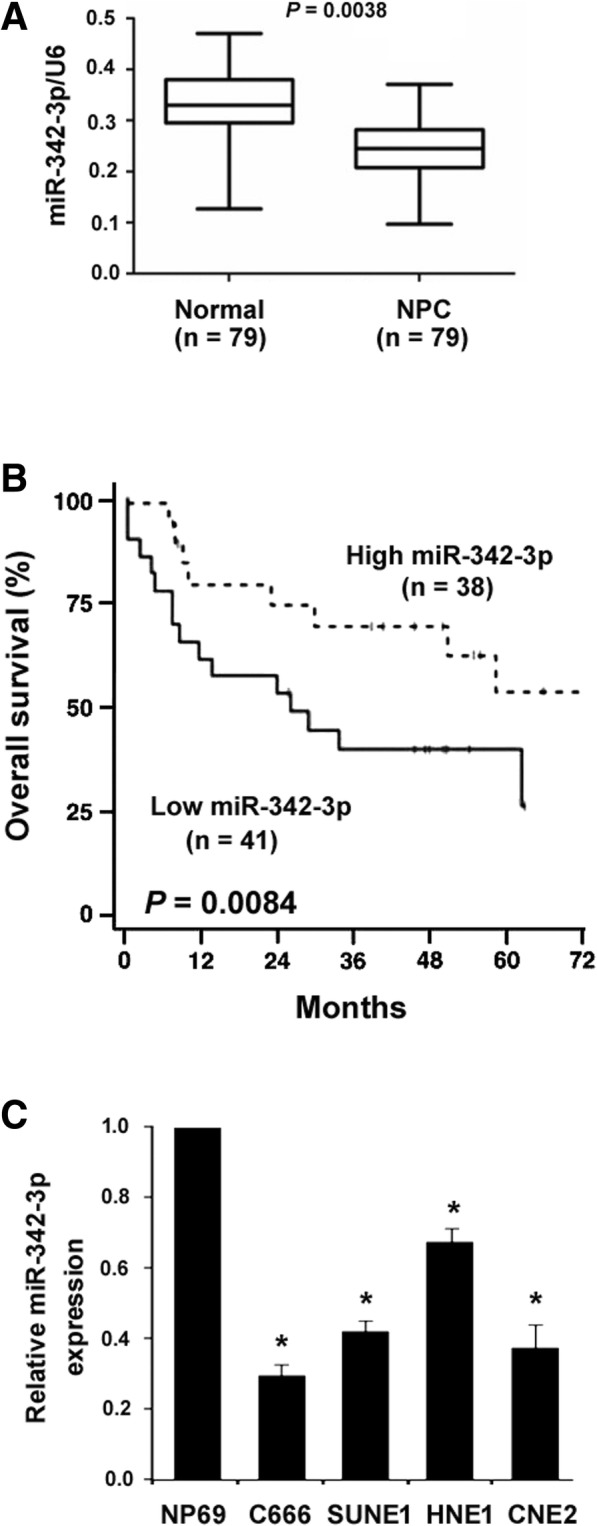
miR-342-3p is downregulated in NPC and predicts poor prognosis. **a** Measurement of miR-342-3p levels in 79 paired NPC specimens and adjacent normal nasopharyngeal tissues by real-time PCR analysis. **b** Kaplan-Meier curves for overall survival in the NPC patients with high (*n* = 38) vs. low (*n* = 41) miR-342-3p expression. The differences between the survival curves were determined by the log-rank test. **c** Measurement of miR-342-3p in indicated cell lines. ^*^P < 0.05 vs. NP69 cells

A total of 79 paired non-malignant and NPC specimens were analyzed for miR-342-3p expression. Compared with corresponding normal nasopharyngeal tissues, NPC tissues displayed a significant downregulation of miR-342-3p (*P* = 0.0038; Fig. 1a). According to the median level of miR-324-3p in NPC specimens, the 79 patients were divided into two groups. The prognostic significance of miR-342-3p expression was then evaluated using the Kaplan-Meier method and log-rank tests. It was found that low miR-342-3p levels were significantly correlated with shorter overall survival (*P* = 0.0084; Fig. 1b) of the NPC patients. These results suggest an important role for miR-342-3p in the pathogenesis of NPC.

### miR-342-3p suppresses NPC cell growth and invasion in vitro

**Fig. 2:**
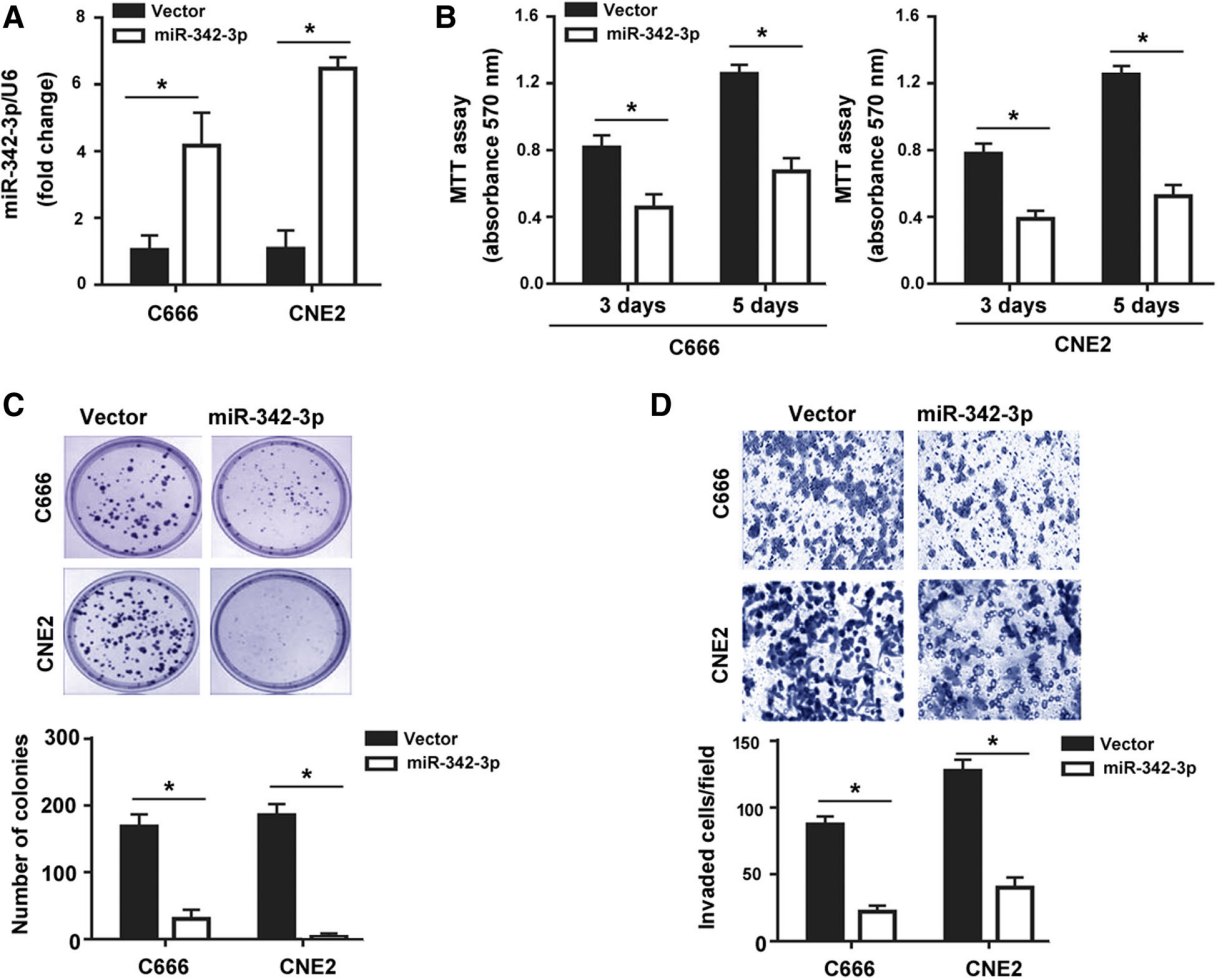
miR-342-3p suppresses NPC cell growth and invasion in vitro. **a** Real-time PCR analysis of miR-342-3p levels in NPC cells transfected with the miR-342-3p-expressing plasmid or vector. **b** Analysis of proliferation by MTT assay in NPC cells transfected with the miR-342-3p-expressing plasmid or vector. **c** Colony formation assay performed in NPC cells transfected with the miR-342-3p-expressing plasmid or vector. *Left*, representative images of colonies. **d** Transwell invasion assay. Ectopic expression of miR-342-3p significantly decreased the invasion of C666 and CN2 cells, compared to control cells. Column: mean of three independent experiments performed in three replicates. Error bar: SD. ^*^P < 0.05

Next, we investigated the effect of restoration of miR-342-3p on the aggressive phenotype of NPC cells. Real-time PCR analysis that NPC cell lines including C666 and CNE2 showed significantly lower levels of miR-342-3p than NP69 non-malignant cells (Fig. 1c). The upregulation of miR-342-3p in both C666 and CNE2 cells stably transfected with the miR-342-3p-expressing plasmid (Fig. 2a). As determined by MTT assay, the number of viable cells was significantly reduced in miR-342-3p-overexpressing cells relative to control cells after culturing for 3 and 5 days (*P* < 0.05; Fig. 2b). Colony formation assay further showed that miR-342-3p overexpression reduced the ability of C666 and CNE2 cells to form colonies (*P* < 0.05; Fig. 2c). To determine whether miR-342-3p affects the invasion of NPC cells, Transwell invasion assay was conducted. The results demonstrated that ectopic expression of miR-342-3p significantly inhibited the invasion of C666 and CNE2 cells by 74 and 68%, respectively (*P* < 0.05; Fig. 2d).

**Fig. 3:**
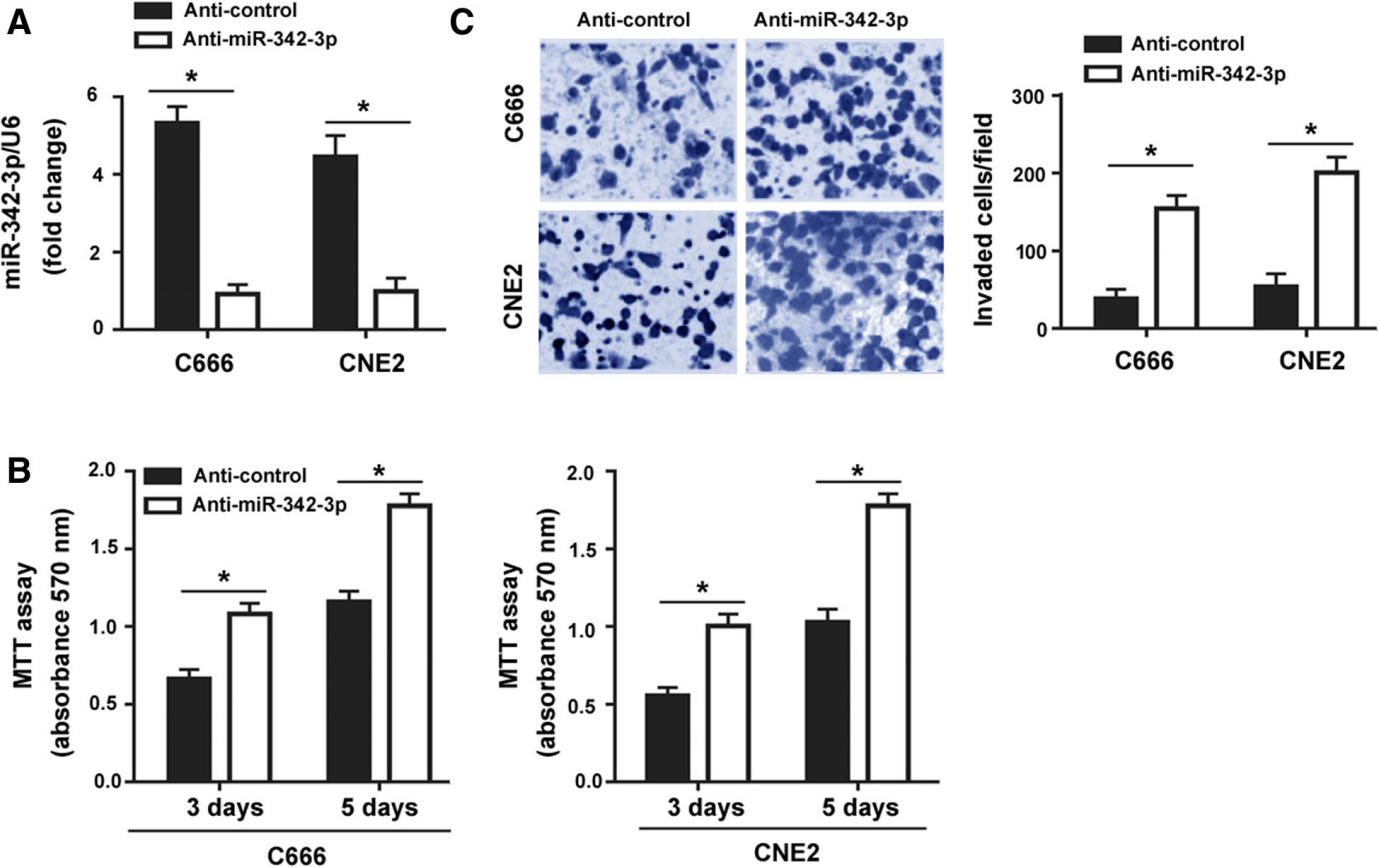
Depletion of miR-342-3p promotes NPC cell growth and invasion. **a** Real-time PCR analysis of miR-342-3p levels in C666 and CN2 cells transfected with control (Anti-control) or anti-miR-342-3p inhibitors. **b** Analysis of proliferation by MTT assay in C666 and CN2 cells transfected with control or anti-miR-342-3p inhibitors. **c** Transwell invasion assay was employed to assess the invasive ability of C666 and CN2 cells transfected with control or anti-miR-342-3p inhibitors. Column: mean of three independent experiments performed in three replicates. Error bar: SD. ^*^P < 0.05

To further confirm the suppressive roles of miR-342-3p, cell proliferation and invasion were examined after inhibition of miR-342-3p. It was found that transfection with anti-miR-342-3p significantly decreased the expression of miR-342-3p (Fig. 3a), which was accompanied by increased cell proliferation (Fig. 3b) and invasion (Fig. 3c) in both C666 and CNE2 cells.

### miR-342-3p restrains the growth of NPC xenograft tumors in vivo

**Fig. 4:**
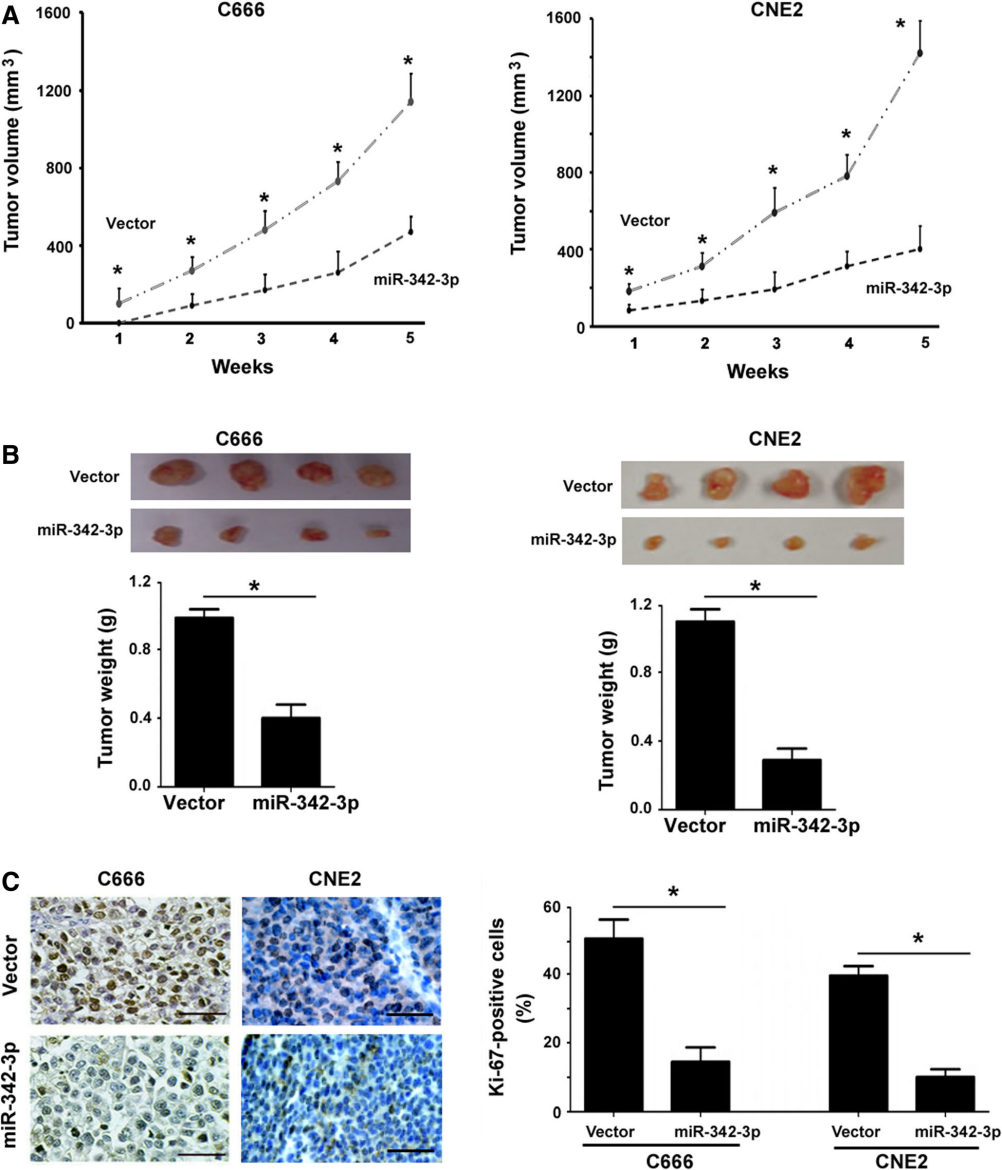
miR-342-3p restrains the growth of NPC xenograft tumors in vivo. **a** Measurement of the growth of xenograft tumors formed by C666 and CNE2 cells stably transfected with the miR-342-3p-expressing plasmid or vector (*n* = 4; error bar: SD). **b** Final tumor weight was determined at 5 weeks after cell injection. Column represents the mean value for 4 mice (error bar, SD). *Top*, macroscopic view of the tumors. **c** Immunohistochemical staining for Ki-67 in the C666 and CNE2 xenograft tumors. Column represents the mean value for 4 mice (error bar, SD). Scale bar = 100 μm. ^*^P < 0.05

Next, we assessed the effects of miR-342-3p overexpression on tumor growth in vivo. All the 4 mice injected with empty vector-transfected C666 cells developed a palpable tumor 1 week later, whereas the onset of tumor development was delayed by 1 week in the corresponding miR-342-3p overexpression group (Fig. 4a). Over an observation period of 5 weeks, the tumor growth rate was slower in the miR-342-3p overexpression group than than in the vector group (*P* < 0.05; Fig. 4a). When empty vector- or miR-342-3p-transfected CNE2 cells were implanted into nude mice, a minor tumor was palpable at 1 week in both the groups (Fig. 4a). However, there was a marked reduction in the tumor growth rate in the miR-342-3p overexpression group, compared to the corresponding vector group. Final tumor weight was reduced in the miR-342-3p overexpression group (0.39 ± 0.07 and 0.32 ± 0.06 g in C666 and CNE2 tumors, respectively) than in the control group (0.88 ± 0.04 and 1.12 ± 0.08 g in C666 and CNE2 tumors, respectively; Fig. 4b). Immunohistochemical staining confirmed that overexpression of miR-342-3p led to a significant decline in the percentage of Ki-67-positive cells in the xenograft tumors (Fig. 4c). These data indicated that miR-342-3p shows growth-suppressive activity in NPC cells in vivo.

### miR-342-3p targets FOXQ1 in NPC cells

**Fig. 5:**
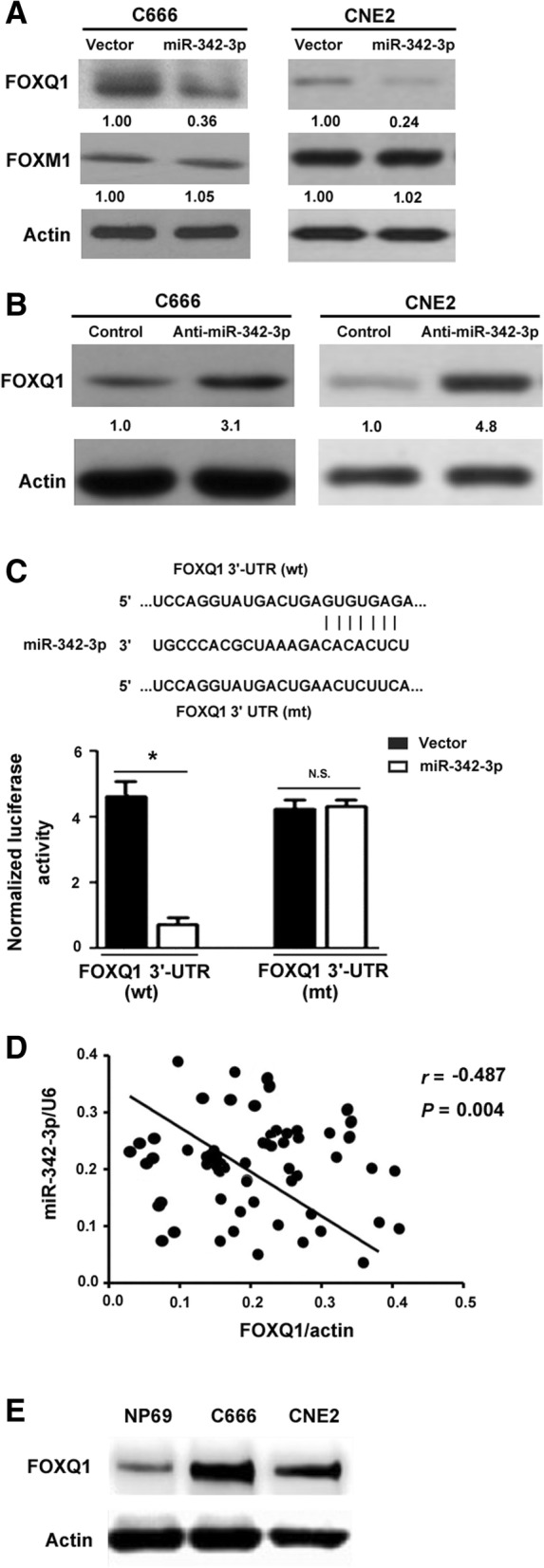
miR-342-3p targets FOXQ1 in NPC cells. **a** and **b** Western blot analysis of indicated proteins in C666 and CNE2 cells transfected with indicated constructs. Numbers represent fold change in protein levels. Data represent mean of three independent experiments. **c** Luciferase reporter assay performed in C666 cells demonstrated that the reporter construct harboring wild-type (wt) *FOXQ1* 3’-UTR but not the mutant (mt) was suppressed by overexpression of miR-342-3p. ^*^P < 0.05. N.S. indicates no significance. **d** Determination of the relationship between miR-342-3p and FOXQ1 protein expression in NPC specimens (*n* = 79) by Pearson’s correlation coefficient analysis. **e** Western blot analysis of FOXQ1 protein levels in indicated cells

It has been documented that both FOXM1 and FOXQ1 serve as two targets of miR-342-3p in colorectal cancer [21]. Given the importance of FOX proteins in NPC development, we tested whether miR-342-3p exerts its suppressive activity through targeting of FOXM1 and FOXQ1. Western blot analysis showed that overexpression of miR-342-3p markedly downregulated the expression of FOXQ1, but had little effect on FOXM1 expression in C666 cells (Fig. 5a). In contrast, delivery of anti-miR-342-3p promoted the expression of FOXQ1 (Fig. 5b) in C666 cells. Similar findings were observed in CNE2 cells (Fig. 5a and b). Luciferase reporter assay showed that overexpression of miR-342-3 p significantly suppressed the wild-type FOXQ1 3’-UTR reporter construct, but not the mutant form (Fig. 5c). We measured the protein levels of FOXQ1 in NPC specimens by Western blotting. Analysis of the relationship between miR-342-3p and FOXQ1 expression in NPC specimens revealed that there was a significantly negative correlation between miR-342-3p and FOXQ1 expression (*r* = − 0.487, *P* = 0.004; Fig. 5d). However, no significant correlation was detected between the expression of miR-342-3p and FOXM1 in NPC tissues (*P* > 0.05). In addition, we found an upregulation of FOXQ1 in both C666 and CNE2 cells compared to NP69 cells (Fig. 5e). These data suggest that FOXQ1 is a direct target of miR-342-3p in NPC.

### FOXQ1 rescues NPC cells from miR-342-3p-induced growth and invasion suppression

**Fig. 6:**
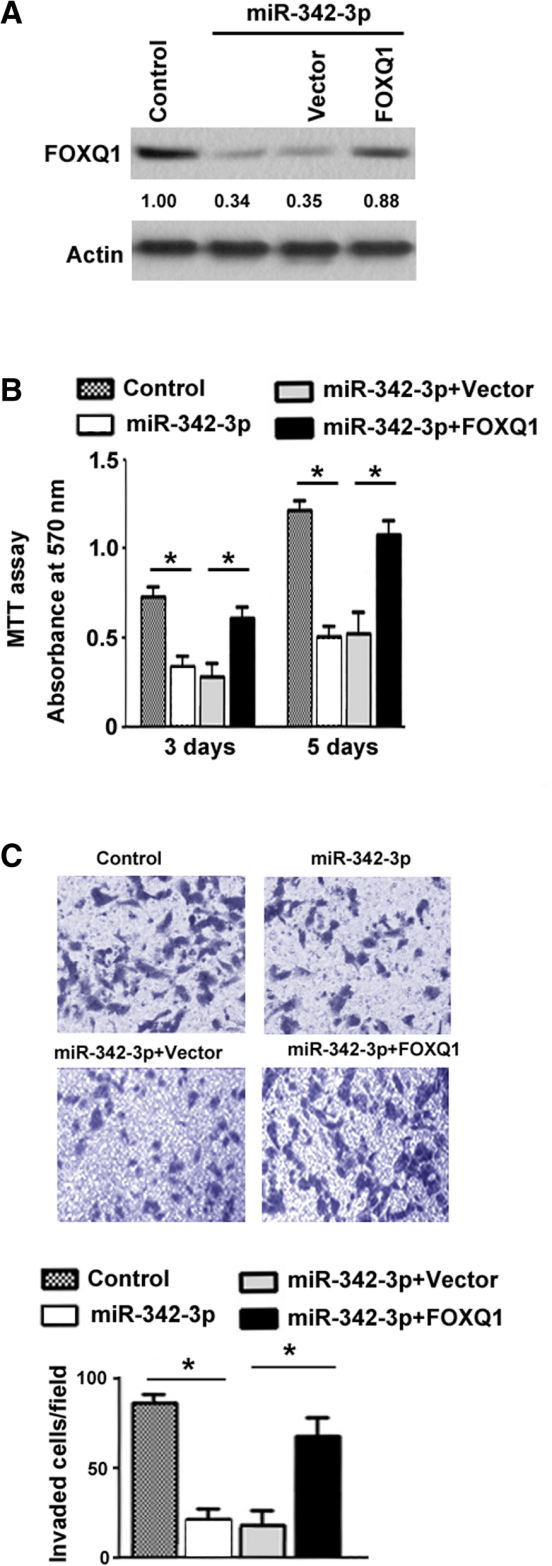
FOXQ1 rescues NPC cells from miR-342-3p-induced growth and invasion suppression. **a** Western blot analysis of FOXQ1 protein levels in C666 cells co-transfected with miR-342-3p- and FOXQ1-expressing plasmid or vector. Numbers represent fold change in protein levels. **b** Analysis of proliferation by MTT assay in C666 cells transfected with indicated constructs. **c** Transwell invasion assay was employed to assess the invasive ability of C666 cells transfected with indicated constructs. Top, representative images of invaded cells. Column: mean of three independent experiments performed in three replicates. Error bar: SD. ^*^P < 0.05

Having identified the downregulation of FOXQ1 by miR-342-3p, we next addressed whether enforced expression of FOXQ1 can rescue the suppressive effect of miR-342-3p on NPC cells. To this end, we overexpressed a miR-resistant variant of FOXQ1 in C666 cells together with ectopic expression of miR-342-3p (Fig. 6a). Overexpression of FOXQ1 significantly restored the proliferation (Fig. 6b) and invasion (Fig. 6c) capacity of miR-342-3p-overexpressing C666 cells. These observations suggest that FOXQ1 mediates the activity of miR-342-3p in the regulation of NPC growth and invasion.

## Discussion

In this study, we showed that miR-342-3p was significantly underexpressed in NPC tissues compared to adjacent normal nasopharyngeal tissues. In line with our findings, the dysregulation of miR-342-3p is also described in colon cancer [14], pancreatic cancer [15], and hepatocellular carcinoma [22]. We further analyzed the relationship between miR-342-3p expression and survival of NPC patients. It was found that downregulation of miR-342-3p had a poor prognostic impact on the survival of patients with NPC. Similarly, low miR-342-3p expression is significant associated with reduced overall survival in patients with hepatocellular carcinoma [22]. These observations favor the idea that miR-342-3p may govern the progression of NPC.

To clarify the biological relevance of miR-342-3p downregulation in NPC, we performed gain- and loss-of-function experiments. The results demonstrated that ectopic expression of miR-342-3p significantly suppressed proliferation, colony formation, and invasion of NPC cells. In contrast, depletion of miR-342-3p led to a significant enhancement of NPC cell proliferation and invasion. In vivo tumorigenic studies confirmed that miR-342-3p overexpression hampered the growth of NPC xenograft tumors. These data indicate that miR-342-3p acts as a tumor suppressor in NPC. In support of our point, several previous studies have documented that miR-342-3p plays a tumor-suppressive role in multiple malignancies including hepatocellular carcinoma [16], colorectal cancer [21], osteosarcoma [23], and small cell lung cancer [24]. However, a recent study has provided evidence that miR-342-3p has no significant impact on the progression of pancreatic acinar carcinoma in a mouse model [25]. Therefore, the functional consequence of miR-342-3p depends on cellular contexts.

It is well accepted that a single miR has the ability to modulate a large number of target genes [11]. Different targets have been identified to mediate distinct biological activities of miR-342-3p [23, 26, 27]. For instance, miR-342-3p regulates lumen formation in mammary gland morphogenesis through repression of ID4 and DNMT1 [26]. This miR shows the ability to induce adipogenesis of mesenchymal stem cells by suppressing CtBP2 [27]. Both FOXM1 and FOXQ1 mediate the tumor-suppressive activity of miR-342-3p in colorectal cancer cells [21]. Similarly, we found that miR-342-3p overexpression caused a significant downregulation of FOXQ1 in both C666 and CNE2 cells. However, the level of FOXM1 remained unchanged in response to miR-342-3p overexpression. The selective repression of FOXQ1 implies its critical role in mediating the activity of miR-342-3p in NPC. Clinical evidence indicates a negative correlation between miR-342-3p and FOXQ1 expression in NPC specimens. Rescue experiments using a miR-resistant variant of FOXQ1 demonstrated that enforced expression of FOXQ1 significantly reversed the suppressive effect of miR-342-3p on NPC cell proliferation and invasion. FOXQ1 functions as an oncogene in colorectal carcinoma [28], gastric cancer [29], and NPC [10]. It has been documented that FOXQ1 can facilitate the metastasis of gastric cancer cells through upregulation of Snail [29]. FOXQ1 is upregulated in NPC and can be regulated by a number of miRs including miR-124 [10] and miR-506 [30]. Our data expand the list of miR regulators of FOXQ1 to include miR-342-3p and confirm the upregulation of FOXQ1 as a consequence of dysregulation of miRs in NPC.

However, it should be mentioned that apart from FOXQ1, other target genes may also be involved in the activity of miR-342-3p in NPC. Genome-wide identification of target genes would be necessary to uncover the mechanism by which miR-342-3p governs the aggressive phenotype of NPC cells. The mechanism underlying the downregulation of miR-342-3p in NPC is still elusive. A previous study has indicated that long non-coding RNAs such as FTX [31] show the capacity to repress the expression of miR-342-3p. The reduced expression of miR-342-3p can also be explained as a result of abberrant methylation of the promoter region of its host gene, EVL [32]. Ongoing studies are conducted to address this issue. Another limitation of this study was the enrollment of patients from a single center.

## Conclusions

In summary, our data demonstrate that miR-342-3p has a poor prognostic impact on the survival of NPC patients. Restoration of miR-342-3p suppresses NPC cell growth and invasion via targeting of FOXQ1 and may have therapeutic benefits in the treatment of this malignancy.

## Abbreviations

3′-UTR: 3′-untranslated region.
FOX: forkhead box
miRs: microRNAs
NPC: nasopharyngeal carcinoma
